# 

**Published:** 1874-10

**Authors:** 


### Notice to Subscribers.

With this number, and the next, we shall send out our bills for subscription. The recent change which has been made in the management ot this JOURNAL, make it desirable that our subscribers should be prompt in their payments. By the new postal law, after the first of January, we shall pay the postage on all our Journals sent to subscribers; we shall not however increase the price of subscription, but shall depend rather upon the prompt payment of the present rate. All remittances should be made to the Assistant Editor, Dr. E. N. BRUSH, No. 8 South Division street, to whom all communications concerning the Journal should be addressed.

### Books and Pamphlets Received.

A Practical Treatise on the Diseases of Women By T. Gaillard Thomas, M. D., etc. Fourth Edition, thoroughly revised with illustrations. Philadelphia: Henry C. Lea, 1874. Buffalo: T. Butler & Son.

Therapeutics and Materia Medica. A Systematic Treatise on the Action and Uses of Medicinal Agents, including their Description and History. By Alfred Stille, M. D., etc. Fourth Edition, thoroughly revised and enlarged. In Two Volumes. Philadelphia: Henry C. Lea, 1874. Buffalo: T. Butler & Son.

Essentials of the Principles and Practice of Medicine. A Handbook for Students and Practitioners. By Henry Hartshorne, A. M., M. D, etc. Fourth Edition, thoroughly revised. Philadelphia: Henry C. Lea, 1874. Buffalo: T. Butler & Son.

Clinical Lectures on Various Important Diseases; being a collection of the Clinical Lectures delivered in the Medical Wards of Mercy Hospital, Chicago. By N. S. Davis, A. M, M. D., etc. Edited by Frank H. Davis, M. D. Second Edition. Philadelphia: Henry C. Lea, 1874. Buffalo: Theo. Butler & Son.

Clinical Lectures on Diseases of the Nervous System. By Wm. A. Hammond, M. D., etc. Reported and Edited by T. M. B. Cross, M. D., etc. New York: D. Appleton & Co. Buffalo: Martin Taylor.

Infant Diet. By A. Jacobi, M D., etc. Revised, enlarged and adapted for popular use. By Mary Putnam Jacobi, M. D. New York: G. P. Putnam's Sons, 1874.

The Building of A Brain. By Edward H. Clarke, M. D. Boston: Jas. R. Osgood & Co., 1874. Buffalo: Herger & Ulbrich.

Croup in its Relations to Tracheotomy. By J. Solis Cohen, M. D. Philadelphia: Lindsay & Blakiston, 1874.

The Physicians Visiting List for 1875. Philadelphia: Lindsay & Blakiston.

The Breath and Diseases which give it a Fetid Odor. With directions for treatment. By Joseph W. Howe, M. D., etc. New York: D. Appleton & Co., 1874. Buffalo: Martin Taylor.

Transactions of the Medical Society of the State of Pennsylvania for 1874.

**THE BUFFALO**

**Medical and Surgical Journal**

PUBLISHED MONTHLY,

Containing Original Articles, Reports of Medical Societies and Hospitals, Editorial, Reviews. Correspondence, Army News, etc., etc ; including the usual variety of Medical Periodical Publications. Specimen copies sent on application.

Address Buffalo Medical & Surgical Journal, Buffalo, N. Y.

TERMS OF SUBSCRIPTION, - - $3.00 PER YEAR, IN ADVANCE.

